# Death of Mrs Sholars

**Published:** 1897-08

**Authors:** 


DEATH OF MRS SHOLARS.—The JOURNAL regrets to learn, by telegraphic dispatch, of the death of Mrs. Sholars, wife of our esteemed friend, Dr. S. W. Sholars, of Orange, Texas. Mrs. Sholars, with her baby and nurse, was driving in a buggy, and, reaching the ford in a stream which they had to cross, the nurse got out of the buggy to let down the check rein so that the horse could drink. Mrs. Sholars drove a little way into the water, when her horse became frightened at a large fish and shied off into deep water. The nurse snatched the child out of the buggy, saving its life and her own; but Mrs. Sholars was drowned. The JOURNAL extends its sympathy to the bereaved husband.

---

THE PROTEST against attempting to organize a medical college at Dallas is signed by a large number of reputable physicians, but as we observe a statement in Dr. Armstrong's diatribe to the effect that "quite a number of respectable physicians had been roped into the scheme,"—roped in—yes, that was the word, we consulted a directory to see what proportion of the profession of Dallas had not signed the circular. We find, in numbers, less than one-half of them signed it. Still, it is not to be inferred that all who did not sign it have been "roped in." Yet it shows that the sentiment against such an enterprise is not unanimous. Surely it will not be denied that the friends of such an enterprise have a right to differ with their colleagues on the subject,—as a matter of policy,—and their opinion is entitled to respect. We do not know precisely to whom Dr. Armstrong refers when he speaks of the "original promoters," and says they are "not worthy of recognition." We have elsewhere defended Drs. Rosser and Milliken against the assertion. We observe that the names of Drs. Leake, Ashton, Chilton, Stout, Du Pre, and many others do not appear to the circular. If these or any of these gentlemen are referred to as having been "roped in," and as "unworthy of recognition"—it is unnecessary to say one word in refutation of the charge; they are all too well and favorably known to require it. Still, we regard it as unfortunate if they have allied themselves with any movement to establish a medical college at Dallas. The attempt must be a foregone failure, as was that at San Antonio some years ago, to which the very best men in San Antonio lent their sanction and their names, Cupples amongst them. This attempt at San Antonio alone, shows that there are men "entitled to recognition" who take issue with us and with the signers of the circular letter as to multiplying medical colleges. There is no room for—nor need of—a medical college at Dallas; and the attempt and failure to establish one would bring the whole State into ridicule.

